# Improving dental student confidence through the use of simulated patient cases

**DOI:** 10.1111/eje.12867

**Published:** 2022-11-07

**Authors:** Jonathan E. Marsden, Stephen P. Deboo, Matt Cripps, Nicholas N. Longridge, Michael Aspden, Kathryn Fox

**Affiliations:** ^1^ School of Dentistry University of Liverpool Liverpool UK

**Keywords:** case‐based learning, e‐portfolio, reflection, simulation, student confidence

## Abstract

**Introduction:**

This study investigates whether student confidence could be improved through the use of simulated patient case‐based scenarios.

**Materials and Methods:**

Students in their 4th and 5th year of undergraduate study completed an online e‐portfolio workbook consisting of activities related to five simulated patient cases. Following completion of the relevant learning activities within the e‐portfolio, students then viewed a tutor case presentation video and attended simulated clinic sessions to complete corresponding exercises involving 3D‐printed teeth related to the case. Subsequently, students undertook online self‐reflection and goal‐setting activities to aid their development. An anonymous questionnaire was distributed to all participants to gain insight into the themes of student confidence and learning support. The Cronbach's Alpha coefficient was calculated for both sections of the student questionnaire. The values for “student confidence” and “learning support” were α = 0.91 and α = 0.87, respectively.

**Results:**

There was a total of 89 valid student responses to the questionnaire (65%). A Spearman's correlation of *r*_s_ = 0.50 (*p* < .001) suggests a tentative causative correlation between the learning support offered through the simulated patient cases and student confidence. There was a positive directional relationship between engagement with the learning support of the simulated patient cases and student confidence scores.

**Conclusion:**

Student confidence increased following the completion of simulated patient cases and students found the learning support offered beneficial to their development. This learning intervention has the potential to improve student self‐efficacy, develop contextual competency and facilitate reflective practice. Simulated patient cases may be a useful precursor or adjunct to traditional patient clinics.

## 1 INTRODUCTION

A student clinician's development and success hinge largely on the ability to retain a large amount of information and apply it to a myriad of clinical situations. Once the learner has begun to gain the basic technical skills required to be a “safe beginner,” the difficulty is then increased through the introduction of patient treatment, with all the accompanying intricacies and complexities. This leap from learning clinical skills on a training simulator in a laboratory to trying to replicate and adapt these skills to a real‐life patient is often referred to as the clinical transition gap and for many students can be a source of much apprehension and self‐doubt.^1^

Self‐confidence is defined by the Oxford English Dictionary as “confidence in one's abilities, qualities and judgements.”^2^ Student confidence in the provision of clinical care is considered a key learning outcome for undergraduate dental students.^3^ It has been shown to play a vital role in educational development and correlates with successful academic performance.^4^, ^5^ Educational experiences that support student development, and are appropriately related to the learner's experiences, are essential in developing competence and confidence.^6^, ^7^, ^8^ Such constructivist approaches are known to support learner self‐regulation, particularly so when educator support is offered through each phase of planning, action and reflection.^6^, ^9^ A review of the literature shows that preclinical simulation is routinely used in healthcare education^10^ and there are several methods possible for designing appropriate simulation tasks. Following consideration of the fundamentals of the 12 best practices of simulation‐based medical education^7^; the 11 components of healthcare simulation‐based curriculum design^11^ and the 8 phases of simulation‐based learning activity cycle^12^ the authors used a constructivist approach with scaffolded learning based on an interpretation of Vygotsky's Experimental Theory. This interpretation of theory as described by Higgins et al.^10^ is set in a socio‐cultural context and is commonly used in researching simulation training within healthcare. It is based on the premise that if learners are immersed in an environment where they experience others undertaking skills at a higher level than themselves, they would enter the “Zone of Proximal Development” and develop the cognition to master such skills themselves.^13^, ^14^ This developmental learning should also encourage intrinsic motivation^15^ through reflective practice^16^ on concrete learning experiences, and goal‐setting with a view to developing self‐regulation. To facilitate effective self‐reflection the importance of consistent and timely coaching‐style feedback was also understood.^17^, ^18^

During a UK dental undergraduate's 4th and 5th year of study, a large proportion of time is usually spent in patient treatment clinics. In March 2020, the COVID‐19 pandemic significantly impacted normal dental undergraduate teaching. In particular, due to social distancing measures imposed by the UK government and the suspension of all non‐urgent dental activity, the ability for students to take those skills learned in a simulated environment and transfer them to patient care was lost.^19^

The challenge faced during this period was how students could continue to develop clinical skills in a meaningful way, without any or limited direct patient contact.^20^ It was believed that this break in clinical activity could potentially negatively impact the confidence that undergraduate students had in their abilities.

Many institutions running clinical courses during this period faced the same dilemma of how to provide quality teaching in a time of social distancing. With the recognised benefits of online learning,^21^ we believed a hybrid learning model would deliver the learning and competency required, through contextualised simulation to best prepare students for the reintroduction of patient care as part of the undergraduate revalidation process.

This study took place within The University of Liverpool School of Dentistry, where learning resources are normally delivered through lectures, seminars, laboratory simulated sessions and patient clinics with an emphasis on active learning, via case‐based scenarios.

The primary aim of this study was to assess if student confidence could be improved through the learning support of simulated patient cases delivered in the environment of conventional adult dental care clinics, utilising a multi‐modality approach with fictitious patient scenarios, online e‐portfolio workbooks and skills training with the aid phantom head simulators and 3D printed teeth. As a secondary research aim, we also investigated the student perceptions of the simulated patient cases and discuss how the benefits of this approach may be of use in the future.

## 2 MATERIALS AND METHODS

This observational cohort study investigated the correlation between the student learning support offered, through the delivery of simulated patient cases, and students' confidence. For the purposes of this study the term “Simulated Clinic” refers to students performing technical and non‐technical skills on a phantom head simulator in a traditional clinical environment.

### 2.1 Production of the simulated patient cases

**FIGURE 1:**
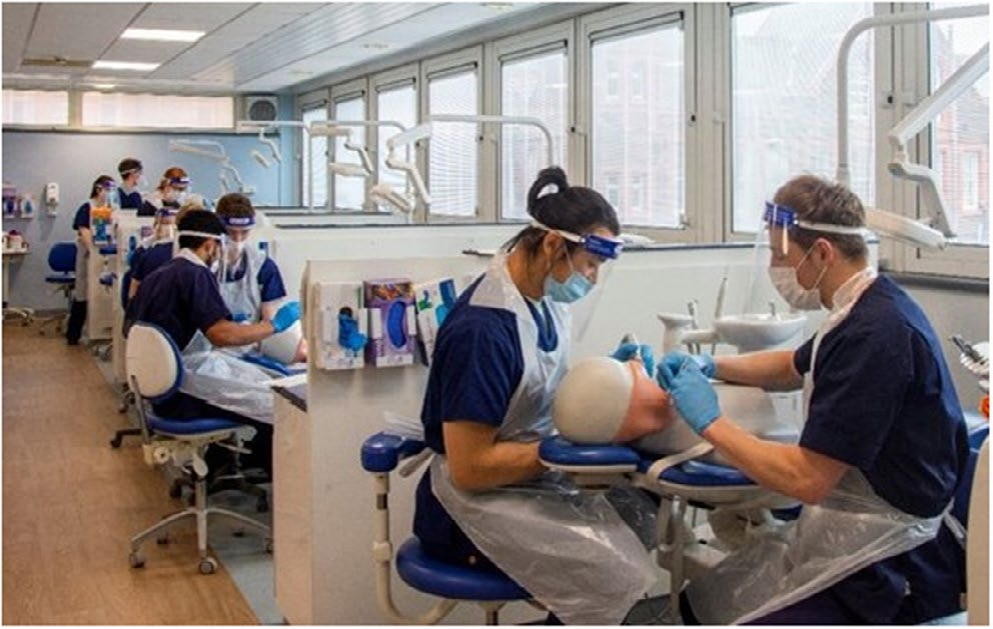
A Photograph was taken by the authors of undergraduate students completing simulated clinical activities in a traditional patient care setting.

**FIGURE 2:**
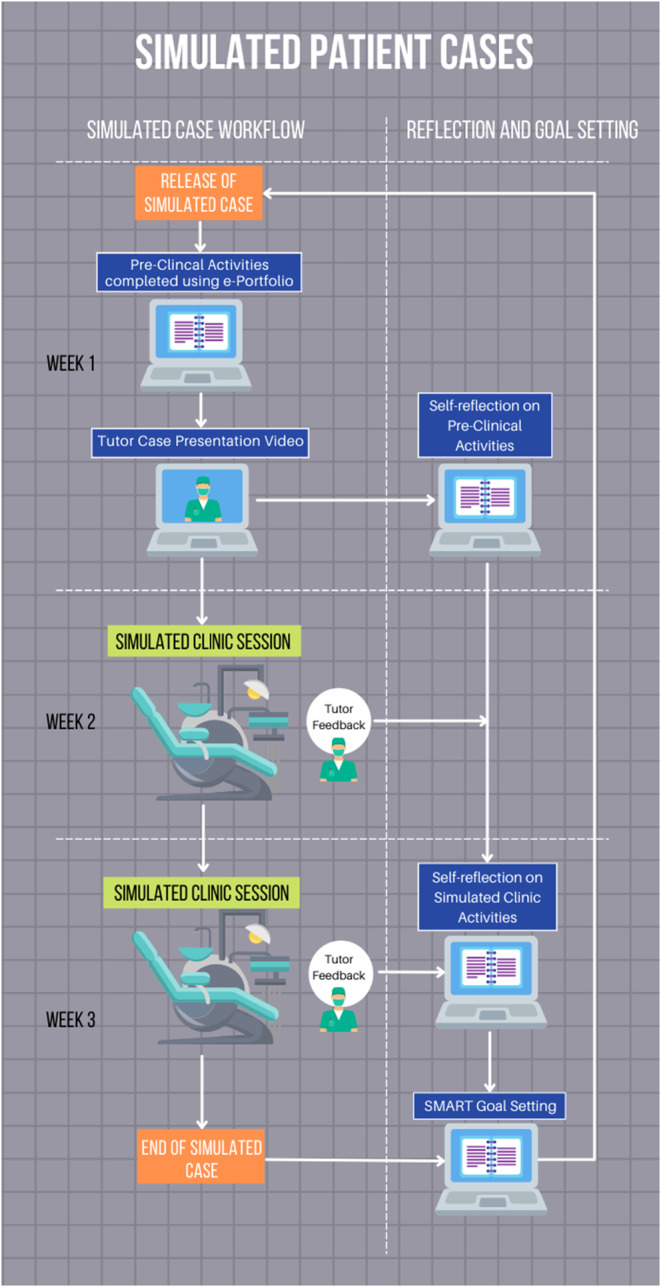
Overview of the simulated patient cases process and the relationship to the self‐reflection, feedback and goal setting.

The 4th‐ and 5th‐year dental students were provided with a series of five simulated patient cases over 14 weeks. A summary of a representative example is shown in Table 1. Clinical members of staff had access to a staff training document regarding the cases. The cases consisted of simulated pre‐clinical, clinical and post‐clinical activities, which were designed to cover a range of skills. The pre‐ and post‐“simulated clinic” activities were completed remotely online, whilst the supervised simulated clinic activities were undertaken using high‐fidelity training models within the normal clinical environment (Figure 1). Self‐reflective activities and subsequent developmental goal‐setting featured heavily throughout all aspects of each case. An overview of the student workflow for the simulated patient cases is shown in Figure 2.

**TABLE 1:** A Summary of a representative simulated patient case example.

Representative example case summary	
Patient details	21‐year‐old, male
Relevant medical history	Allergy to penicillin, seasonal rhinitis. No prescribed or self‐prescribed medication
Presenting complaint	Nil, would like a “routine check‐up”
Past dental history	Last attended 3 years ago. Previous regular attender. Had fixed orthodontic appliances when younger. Not dentally anxious
Social history	Recently moved back to parent's house after completing a university degree, works part‐time in a restaurant
Oral hygiene regime	Manual toothbrush twice daily, no interdental cleaning aids, uses a herbal toothpaste and thinks it does not contain fluoride
Clinical findings summary	Nothing abnormal was detected extra‐orally or on soft tissue examination Cavitated carious lesions UR6, UR3 and UR3. Retained LLE.
BPE	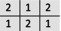
Special investigations provided	Right and left Horizontal bitewing radiographs. Right horizontal radiograph is shown in Figure 4.
Index of multiple deprivation	Overall patient neighbourhood is 40% the most deprived in the country. Within the 20% most deprived neighbourhoods in terms of health deprivation.

### 2.2 Pre‐clinical activities

**FIGURE 3:**
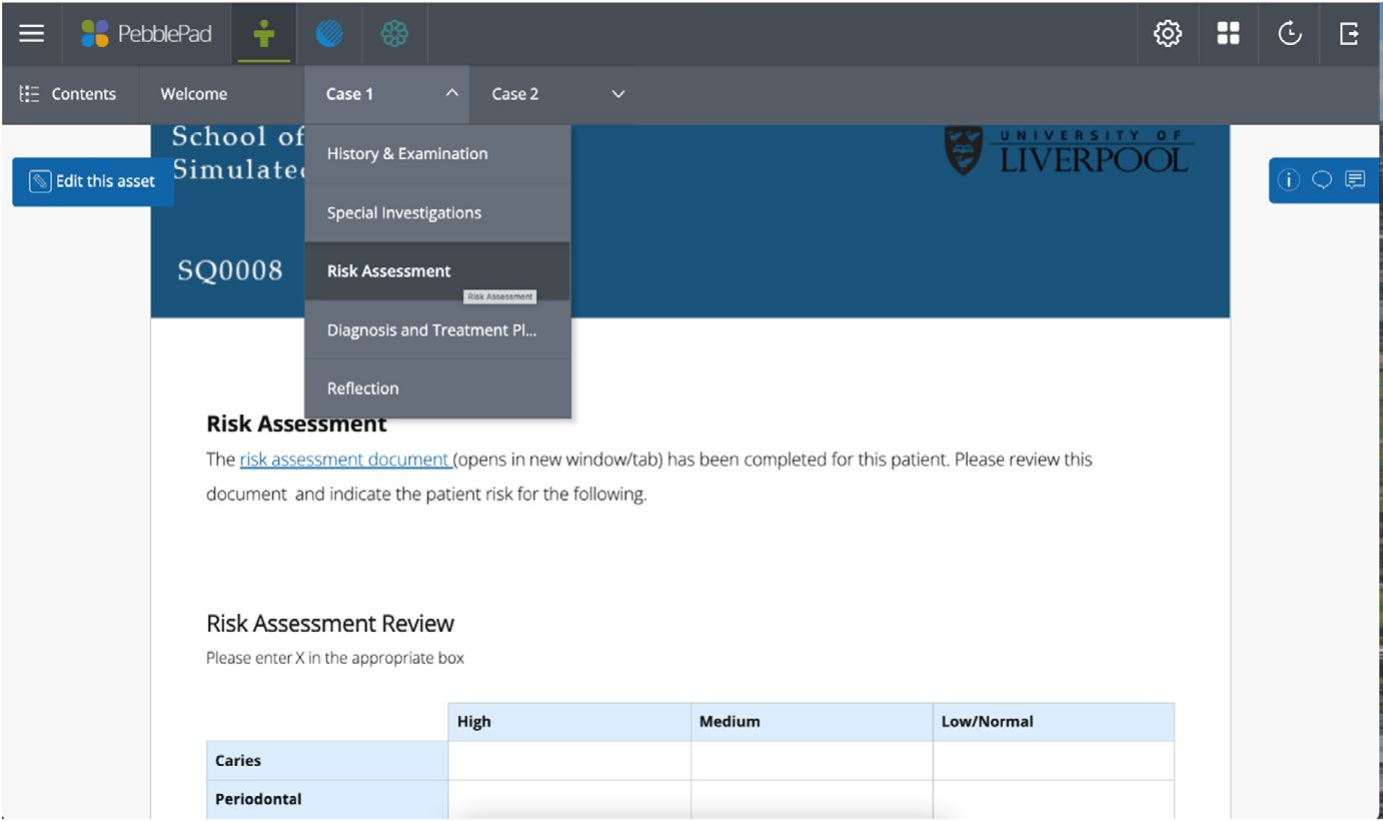
A Screenshot of the e‐portfolio, showing the risk assessment section of case 1, and the link for case 2.

The pre‐clinical activities were released to students via an online e‐portfolio software, PebblePad (Telford, UK), as shown in Figure 3. The e‐portfolio workbook for each simulated patient case was divided into five sections; History & Examination, Special Investigations, Risk Assessment, Diagnosis & Treatment Planning and Reflection. Students worked through each section in order. Information such as the medical history and clinical findings were delivered using replicas of the standard clinical proformas used within the hospital trust. Following completion of the pre‐clinical activities, students gained access to a tutor case presentation video. This was presented asynchronously as a pre‐recording of an experienced clinician talking through appropriate answers for the case within the e‐portfolio. Case presentation videos included supplemental learning resources with voice‐over and typically lasted between 15 and 20 min.

### 2.3 Simulated clinic

Simulated clinical activities were undertaken in the Restorative Dentistry Clinics within The Royal Liverpool University Dental Hospital. As part of the management of the case, students were required to present their “simulated patient” to the clinical tutor at the commencement of the session, to replicate the normal “safety huddle.”^22^ Staff‐student discussions included medical co‐morbidities, diagnoses and treatment care plans for each case based on the pre‐clinical activities and following review of the tutor case presentation video. As cases required multiple treatment items, students were required to modify their case presentation based on completed and planned treatment for each session. Students also completed the relevant simulated clinical procedural records for the interventions provided, including internal referral proformas, consent forms and prescription writing in line with their treatment plan.

Students then individually worked sequentially through their self‐devised treatment plans, overseen by a registered clinician to confirm the treatment plans were clinically appropriate. The treatment was carried out on high fidelity models (Frasaco® GmbH, Tettnang, Germany) and where possible, the clinical presentation of the dental defects within the case was replicated on 3D printed teeth for example simulated dental decay or trauma was added where appropriate to provide a more immersive and realistic learning experience.

### 2.4 Customised high‐fidelity 3D printed models

**FIGURE 4:**
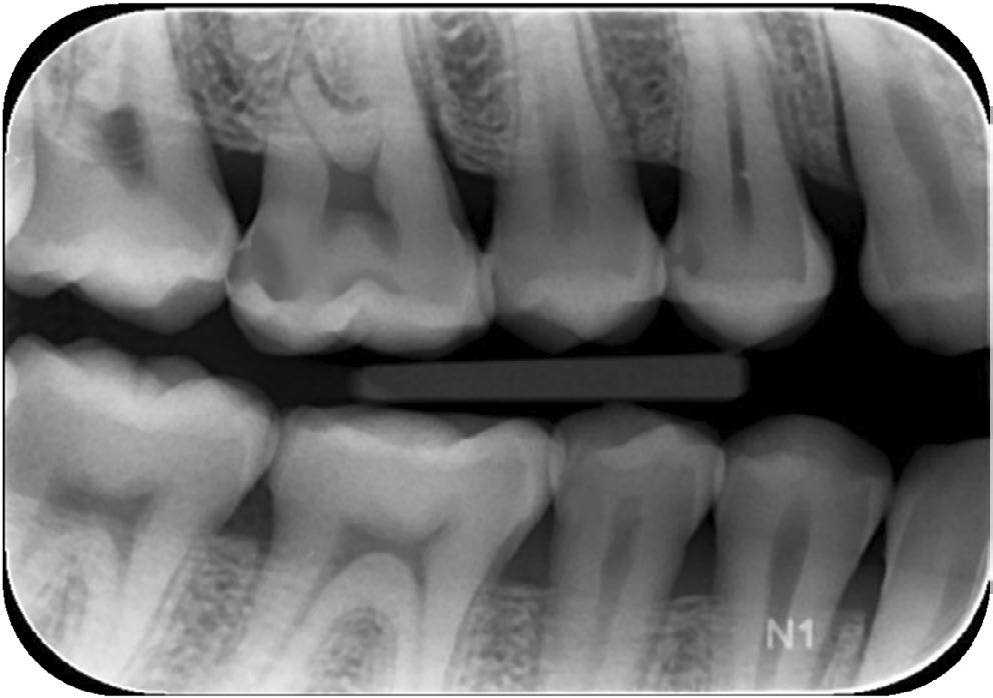
Right horizontal bitewing radiograph, showing carious lesions present in the upper right first permanent molar and upper right first permanent premolar, requiring operative intervention.

**FIGURE 5:**
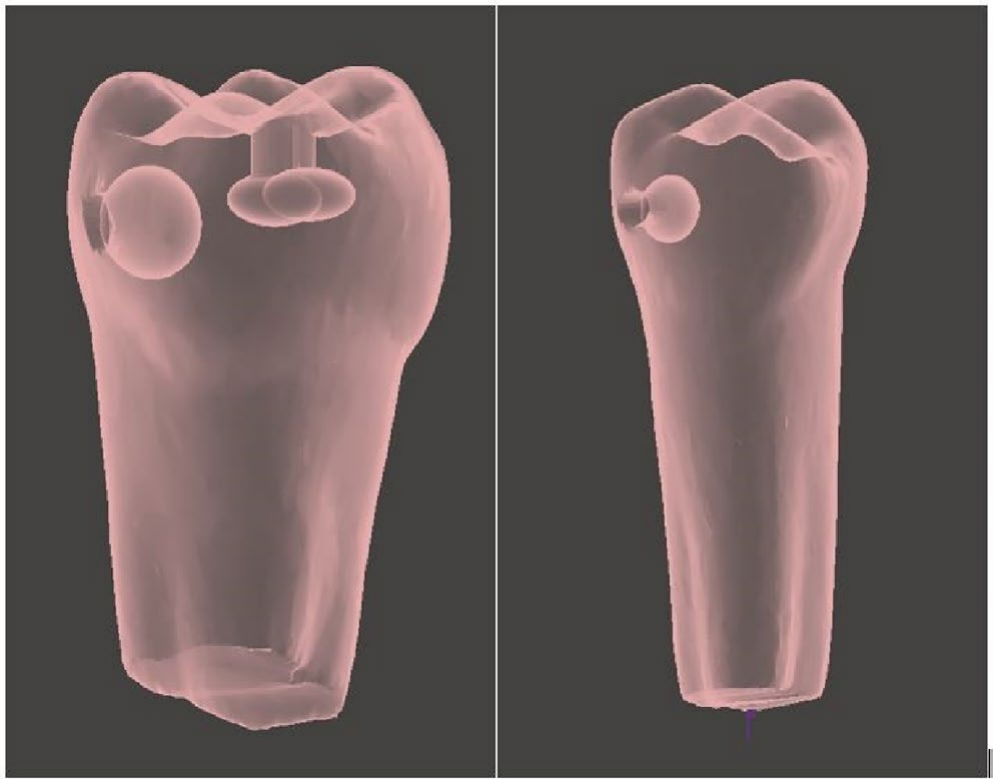
3D Digital rendering of carious lesions shown in Figure 4.

Following the selection of patient cases for simulation, radiographs were collected and visually analysed for carious lesion location and extent (Figure 4). The corresponding typodont tooth (Frasaco®, GmbH) was scanned using a high‐resolution desktop blue light model scanner, Vinyl HR scanner (smart optics Sensortechnik, GMbH, Bochum, Germany). A Standard Triangle Language (.STL) file was then imported into Meshmixer® (Autodesk, Inc.®) to be made watertight and then transferred to TinkerCAD® (Autodesk, Inc.®) a free, online software programme in which the carious lesion design took place. Anatomical sites were matched to the radiographs identified and a range of oval and free‐form primitive shapes were boolean subtracted from the .STL file as appropriate (Figure 5).

As low‐force stereolithography technology was to be used for tooth production, all carious lesions were designed as cavitated (i.e., perforated through the enamel layer to enable the resin to drain during printing). To mimic the destructive penetration of caries along the amelo‐dentinal junction and into dentine, internal lesion dimensions at an appropriate depth were always greater than the extent of enamel penetration.

**FIGURE 6:**
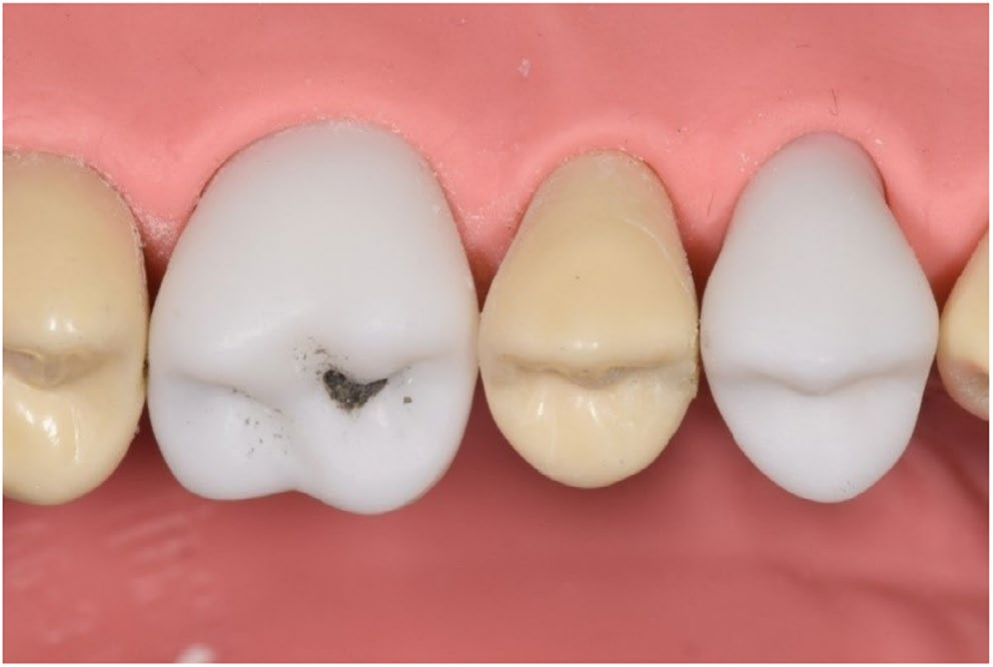
High fidelity model (Frasaco®, GmbH) with customised 3D printed teeth in situ.

Prototype teeth were additively manufactured using low‐force stereolithography on a Form 3B printer (FormLabs Inc.) in white resin. Post‐production wash and cure were performed following manufacturer's instructions in the Form Wash and Form Cure, respectively (FormLabs Inc. ). In brief, an automated wash cycle specific to white resin was undertaken in 99% isopropyl alcohol prior to additional curing with 405 nm blue light with heat. Print supports were removed with plyers and parts were inspected at 8× magnification using a direct operating microscope. Prototypes were trialled, prepared and restored by multiple clinicians prior to upscaling. Simulated dental caries was added post‐production using a mixture of knotting solution (Rustins Ltd), black food colouring (Dr Oetker) and dental stone delivered using a blunt‐end, 27G needle. Screw holes were self‐tapped and teeth were screwed into a standard full arch model, ready for use by undergraduates during simulated clinical activity (Figure 6).

### 2.5 Self‐reflection, feedback and goal setting

Self‐reflection, feedback and goal setting was predominant theme throughout the simulated patient cases, the impact of which has been discussed in papers such as Locke and Latham's “practically useful theory of goal setting.”^23^ These themes were incorporated into simulated pre‐clinical, clinical and post‐clinical activities (Figure 2). Students initially worked independently through the e‐portfolio, which contained a proforma for self‐reflection. The students also had access to the tutor case presentation video to help reflect on their performance in completing the pre‐clinical activities, identify areas for development and detail a plan to address any identified learning needs. To supplement the case discussion video and facilitate consolidation of previously covered material, learning resources pertinent to the case were also circulated, such as national guidance documents and peer‐reviewed journal articles.

During face‐to‐face simulated clinic sessions, tutors could access each student's e‐portfolio and would, through discussion with the student, provide feedback verbally on pre‐clinical activities as well as their performance during the simulated clinical exercises carried out during the session. This feedback was also recorded using LiftUpp®, a digital education software development platform. Following each simulated clinic session, students completed the relevant post‐clinical self‐reflection template within the e‐portfolio, using Rolfe's reflective model (What? So What? Now What?).^24^ Students were familiar with this model due to prior use within their academic studies. Students were then encouraged to formulate a personal development plan according to the Specific, Measurable, Attainable, Relevant and Time‐bound goals framework,^25^ for which a template was available.

Engagement with the e‐portfolio was formatively assessed, and students identified with insufficient engagement were contacted to determine if any barriers to learning could be identified and appropriate support offered.

### 2.6 Study participants

The target population for this study was 4th‐ and 5th‐year undergraduate students studying the Bachelor of Dental Surgery programme at the School of Dentistry within the University of Liverpool. All students who had completed the simulated patient cases were invited to take part in the questionnaire, a potential of 135 students.

### 2.7 Questionnaire

A questionnaire was devised by two researchers and consisted of three sections; learning support, student confidence and open questions. The first and second sections consisted of closed questions utilising 5‐point Likert scale responses although with different agreement response anchors between Section 1 and 2, as shown in Table 3. A similar five‐point semantically anchored Likert scale has been used before to assess self‐efficacy and more specifically confidence in undergraduate dental students.^26^, ^27^, ^28^ The third section contained open questions where students had the opportunity to express their views in greater detail and suggest improvements or ideas for future simulated patient cases (shown in Table 2).

This questionnaire was subject to internal peer review prior to release. The anonymous questionnaire was created using Microsoft® Forms (Microsoft Corporation) and distributed to all eligible students via email and a link was made available via the virtual learning environment. Although anonymous, a student university login was required to access the questionnaire to prevent duplicate responses.

**TABLE 2:** The open questions included in the questionnaire.

Open questions
Please provide comments to explain or clarify your ratings above. Comments may be related to teaching delivery, organisation, clinical sessions or anything else which would help us to improve the Simulated Cases going forwards. Please be as concise as possible.
Do you feel the Simulated Cases offer any advantages for your development compared with treating patients? Please provide comments to support your answer.
Please provide comments on the 3D‐printed teeth, such as, how they aided your development and if you feel they accurately replicate natural teeth and caries.
Please list up to three ways in which the Simulated Clinical Cases could be improved.
Please list any ideas you have for future Simulated Clinical Cases, including 3D‐printed elements.

### 2.8 Ethical considerations

Ethical approval was granted by the University of Liverpool Ethics Committee (approval number 5402). Participation in the questionnaire was voluntary, and participants were not identifiable from the data collected. Prior to recruitment, potential participants were provided with written information relating to the study and consent was obtained for participant responses to be included in the research.

### 2.9 Quantitative data—Statistical analysis

The collected data were interpreted using JASP (Version 0.14.1) and the closed questionnaire questions consisted of two main themes, “student confidence” and “learning support.” To analyse the internal consistency of the questionnaire, Cronbach's Alpha coefficient was calculated for items within the two main themes. As separate response scales were used, these two sections lacked unidimensionality. The value for Cronbach's Alpha coefficient can range from 0 to 1 and is most valuable when reported in relation to single‐construct scales rather than when given for several constructs simultaneously.^29^ Acceptable values were calculated for “student confidence” and “learning support,” α = 0.91 and α = 0.87, respectively, demonstrating a high level of internal consistency reliability.

### 2.10 Qualitative data—Thematic analysis

Two team members (JM and SD) independently read through the responses to the open‐ended questions to conduct a thematic analysis primarily utilising a deductive approach, following a researcher‐driven focus aligned with the aims of the study.^30^ Both of these team members were trained in qualitative methodology, and independently coded responses manually. This coding was then compared and through careful discussion analysed for commonalities and differences. These themes were then discussed until a consensus was reached and key quotes were drawn from answers as illustrative examples.

## 3 RESULTS

In total 89 students (65%) completed the anonymous questionnaire. This sample consisted of 45 students in their 4th year and 44 students in their 5th year of study, 69.2% and 61.1% of the total eligible to participate, respectively. The results of the student questionnaire are presented in Table 3.

**TABLE 3:** Frequency distributions of the students' responses to the anonymous questionnaire relating to the simulated cases as a clinical replacement activity.

Items		*N* ^a^	Frequency distribution (%)					Mean rating
Learning support			1^b^	2^b^	3^b^	4^b^	5^b^	
1	The Simulated Cases have helped me to develop my clinical skills	89	1.1	0.0	2.2	37.1	59.6	4.54
2	The Simulated Cases were well organised and ran smoothly	89	2.2	3.4	20.2	46.1	28.1	3.94
3	The online learning resources and patient cases, via PebblePad, were clear, well‐structured and easy to access	89	0.0	0.0	1.1	32.6	66.3	4.65
4	The case‐based structure of the Simulated Cases has been more beneficial for my development compared with undirected practice (i.e. free practice)	89	0.0	0.0	5.6	24.7	69.7	4.64
5	The Simulated Cases replicated real‐world clinical situations well	89	0.0	2.2	10.1	49.4	38.2	4.24
6	The complexity of the Simulated Cases was appropriate	89	0.0	0.0	7.9	46.1	46.1	4.38
7	The Case Discussion videos helped me identify areas in which I need to develop further	89	0.0	0.0	2.2	41.6	56.2	4.54
8	The Simulated Cases have developed my clinical problem‐solving abilities	89	0.0	1.1	10.1	47.2	41.6	4.29
9	The feedback I received from tutors during Simulated Case sessions helped me identify areas where I can develop	89	0.0	0.0	6.7	48.3	44.9	4.38
10	I found the self‐reflection and goal‐setting helpful in supporting my development in areas identified by feedback	89	9.0	6.7	37.1	27.0	20.2	3.43

Student confidence		*N* ^a^	Frequency distribution (%)					Mean rating
			1^c^	2^c^	3^c^	4^c^	5^c^	
11	Having participated in the Simulated Cases I feel more confident to understand a patient's history	89	0.0	2.2	7.9	38.2	51.7	4.39
12	Having participated in the Simulated Cases I feel more confident to select, interpret and report on special investigations	89	0.0	1.1	4.5	43.8	50.6	4.44
13	Having participated in the Simulated Cases I feel more confident to establish a diagnosis and formulate a treatment plan	89	0.0	0.0	3.4	43.8	52.8	4.49
14	Having participated in the Simulated Cases I feel more confident to conduct a patient risk assessment and use this to develop a prevention plan	89	0.0	2.2	6.7	42.7	48.3	4.37
15	Having participated in the Simulated Cases I feel more confident to present a case	89	1.1	4.5	7.9	30.3	56.2	4.36
16	Having participated in the Simulated Cases I feel more confident to perform non‐surgical periodontal therapy	89	2.2	4.5	16.9	36.0	40.4	4.08
17	Having participated in the Simulated Cases I feel more confident to place a dental dam	88	0.0	1.1	2.3	17.0	79.5	4.75
18	Having participated in the Simulated Cases I feel more confident to manage caries	89	1.1	4.5	9.0	40.4	44.9	4.24
19	Having participated in the Simulated Cases I feel more confident to undertake direct restorations	88	0.0	1.1	4.5	39.8	54.5	4.48
20	Having participated in the Simulated Cases I feel more confident to undertake endodontic therapy	89	5.6	11.2	24.7	38.2	20.2	3.56
21	Having participated in the Simulated Cases I feel more confident to undertake fixed prosthodontic treatment	88	1.1	4.5	8.0	42.0	44.3	4.24
22	Having participated in the Simulated Cases I feel more confident to write accurate and detailed clinical records, e.g., laboratory cards, internal referrals, consent forms, prescriptions and patient case notes	89	0.0	1.1	10.1	40.4	48.3	4.36
23	Having participated in the Simulated Cases I expect to perform these skills more confidently in a real‐world situation.	89	1.1	0.0	6.7	46.1	46.1	4.36

^a^ *N* value varies due to the lack of response to some items in the questionnaire.

^b^ Level of agreement: 1—strongly disagree; 2—agree; 3—neutral; 4—agree; 5—strongly agree.

^c^ Level of agreement: 1—definitely not; 2—probably not; 3—maybe or maybe not; 4—probably yes; 5—definitely yes.

### 3.1 Questionnaire Results—Quantitative Data

#### 3.1.1 Student confidence

**FIGURE 7:**
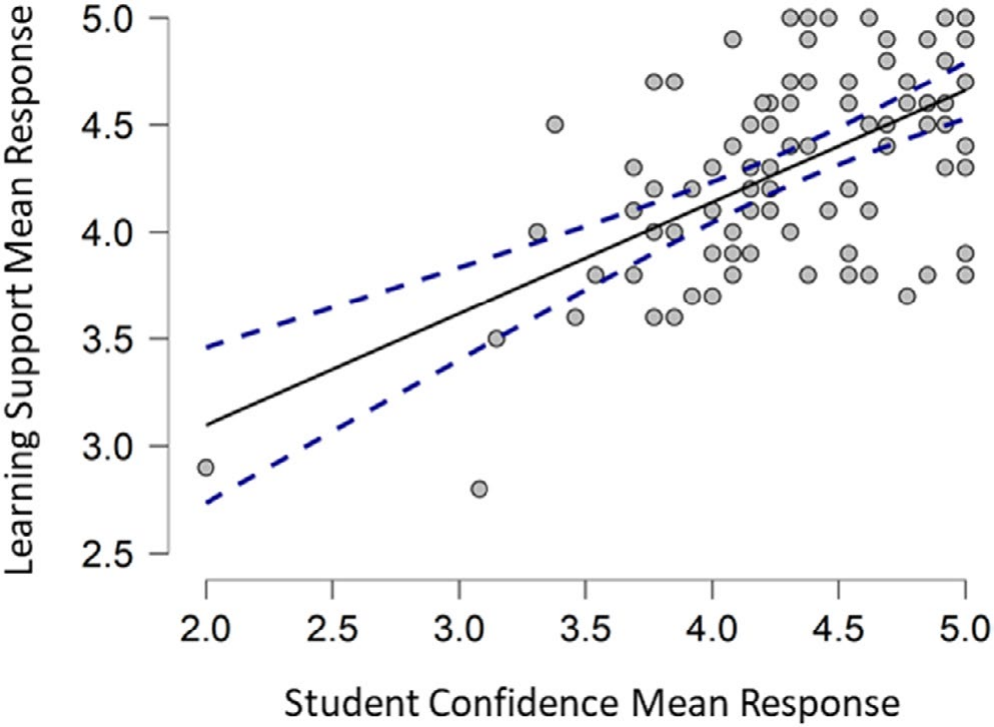
Scatterplot showing the relationship between the mean responses per student for learning support (simulated patient cases) and student confidence. The solid black line is Spearman's correlation coefficient, and the dashed blue line represents the 95% confidence threshold.

As the ordinal data collected were nonparametric, a Spearman's correlation coefficient was performed, *r*_s_ = 0.50 (*p* < .001), this is indicative of a tentative causative correlation between the learning support offered through the simulated patient cases and student confidence—shown in Figure 7. There was a positive directional relationship between engagement with the learning support of the simulated patient cases and student confidence scores.

As demonstrated in Table 3, students felt more confident in non‐technical skills such as understanding a patient's history, the selection and interpretation of special investigation and the ability to diagnose and develop a patient care plan. Students also reported an increase in confidence in technical abilities, with 76.40% of students agreeing they felt more confident to provide non‐surgical periodontal therapy and 85.30% of students agreeing they were more confident to manage a carious lesion appropriately. The largest increase in confidence was seen in the ability to place dental dams and provision of direct restorations, although there was not as marked an increase in student confidence to provide endodontic therapy.

#### 3.1.2 Student perception of learning support

Overall the student perception of the simulated patient cases was overwhelmingly positive. 96.70% of students agreed or strongly agreed the cases had helped to develop their “clinical skills,” whilst 94.40% of students found the contextualisation of the simulated patient cases to have been more beneficial to their development compared to “undirected practice.”

Other notable results from the questionnaire include 87.60% of students felt the simulated patient cases had “*replicated real‐world clinical situations well”* and 92.20% of students agreed that having participated in the simulated patient cases they felt more confident to perform these skills *“in a real‐world situation*.” 97.80% of students also agreed the tutor case presentation videos had helped them to identify areas in which they needed to develop further, whereas only 47.20% of students found the reflection and goal‐setting exercises to be helpful in supporting their development.

### 3.2 Questionnaire results—Qualitative data

#### 3.2.1 Thematic analysis

Thematic content analysis was conducted deductively under two broad themes of “student confidence” and “student perception of learning support,” the latter contained four subthemes of “cased‐based learning and simulated‐clinical sessions,” “online e‐portfolio,” “3D‐printed teeth” and “self‐reflection and goal setting.”

##### Student confidence

Many students reported that they felt more confident in their abilities following the programme of simulated patient cases, particularly in anticipation of the resumption of patient care provision. This included confidence in both technical and non‐technical skills. Students acknowledged the differences in performing tasks on simulators to providing care for a real patient, although students felt the absence of patients created a low‐pressure environment where they were able to develop their skills further and in turn, confidence.

Loved the sessions and feel more confident in my clinical skills than I ever have. (4th‐year student)

I learnt a lot from the tutors, and feel a lot more confident since doing the simulated cases. (5^th^‐year student)

definitely allowed me to build my confidence with a lot less pressure than treating a real patient (4th‐year student)

##### Student perception of learning support

###### Perceptions of case‐based learning and simulated‐clinical sessions

Students reacted positively to the introduction of the simulated patient cases and largely felt they benefitted from having the opportunity to identify and address gaps in their knowledge. Many students also reported the face‐to‐face simulated clinical sessions to be enjoyable and found the feedback from tutors valuable in helping to develop their skills. Some students expressed a desire for more advanced cases involving complex fixed prosthodontics and endodontics, although the simulated patient cases did replicate real‐life situations well they were unable to fully recreate the complexities of true patient interaction.

helped to identify areas that I wouldn't have thought I would struggle with or practiced without directed learning. (5^th^‐year student)

allows you to work through the whole process of a treatment plan and the treatment itself but without the stress of having a patient so allows you to be more relaxed and gain confidence. (4^th^‐year student)

###### Student perceptions of e‐portfolio

The delivery of the e‐portfolio, via PebblePad, and other learning resources were well received by students and they found the content easy to access and navigate. Some students expressed the desire for the ability to upload images of their practical work to the e‐portfolio to aid their reflection.

the organisation of the simulated cases themselves was great and I really liked the PebblePad layout. (4^th^‐year student)

I think overall premise of the Simulated Cases was very good, they were well structured (cases themselves) and PebblePad was a nice way of having to do them in. (4th‐year student)

###### Student perceptions of 3D‐printed teeth

Many students found the 3D‐printed teeth to be a superior learning resource in comparison to normal plastic typodont teeth, appreciating the consistent position and size of the defect corresponding to other learning resources such as the pre‐operative radiograph and clinical images. Students also enjoyed having the same task as their peers, which provided a point of peer discussion. However, students reported that the texture of the 3D‐printed teeth was soft in comparison to natural teeth and that this soft texture lead to challenges during tooth preparation.

Having caries present in the 3D printed teeth was much better as this best replicated how we would be carrying out the restoration in a real‐life patient scenario. (5th‐year student)

Preferred [the 3D printed teeth] much more than the other teeth as you can replicate caries etc. Each prep is personal to a patient. (5th‐year student)

The caries replication was very useful however the teeth are very soft. (5th‐year student)

###### Student perceived value of engagement with self‐reflection and goal setting

Some students perceived engagement with the self‐reflection and goal‐setting area of the e‐portfolio not to be of equivalent educational value and as relevant to their development as other sections of the simulated patient cases.

Personally I didn't find the reflection component that useful as tutors provide feedback on Liftupp and we discuss the feedback then and there. (5th‐year student)

the reflection section was useless to me. (4th‐year student)

## 4 DISCUSSION

Preparing for Practice (2015)^31^ published by the General Dental Council (GDC), sets out the four core competency domains that are required to be demonstrated by a dental student prior to registration. The “*clinical domain*” stipulates that students should be able to “*follow the patient journey, including stages through assessment, diagnosis and patient management*.”

One of the main purposes of this learning modality was to recreate the patient journey and to contextualise the clinical skill activities ordinarily carried out on a simulator. When a student moves from the pre‐clinical to the clinical environment the adaptive capabilities of that student are put to the test. A reliance is also placed on the student to recognise the similarities and differences in the change of environment.^32^ The students' level of competence is traditionally only assessed within the original pre‐clinical setting and a presumption is made about their ability to then replicate this level of performance in the clinical setting. By incorporating a transitional step between the two environments, such as was deployed in this study, competence and confidence in undertaking certain skills can be verified prior to students encountering patients. Self‐efficacy is a “*belief about what a person can do, as opposed to a personal judgement about physical or psychological attributes*.”^33^ For example, a student may feel a high level of self‐efficacy to provide a simple direct restoration but a lower level of self‐efficacy for a more complex procedure, such as endodontic therapy. Bandura argues mastery experiences are the foremost method of forming a strong sense of self‐efficacy,^34^ success reinforces this efficacy whereas failure undermines it. To develop a resilient sense of self‐efficacy students must overcome difficulties through perseverance. Direct involvement with mastery experiences, such as the successful management of a simulated patient case, fosters a feeling of self‐confidence, an antecedent of self‐efficacy.^35^

Reflective practice is believed to encourage deep learning, which over time will lead to improved patient care.^22^ This teaching initiative supports this aim through hybrid active learning^23^, ^24^ and enriched learning‐in‐context,^25^ reflective practice via an e‐portfolio^26^, ^27^ utilising a self‐reflection model^18^ and goal setting for future practice.^17^ Simulated patient cases, in combination with a reflective e‐portfolio appear to facilitate self‐regulated learning^36^ to develop skills and confidence through simulation to increase self‐efficacy.

Of interest was the relatively lower number of students who felt that they agreed or strongly agreed that self‐reflection and goal‐setting supported their development in areas identified by feedback (47%). It could be hypothesised that students may instead prefer to self‐reflect and goal set in other, less formal, ways. A possible explanation for this is that as senior undergraduate students, some may have developed internal reflective practice already and see little benefit in engaging in the formal written process of self‐reflection.^37^ Others may also feel reluctant to honestly reflect on the knowledge it could be read by staff members and may have incorrectly perceived this could be used for assessment or progress purposes. However, it is a requirement of the GDC for those on the dental register to develop a personal development portfolio.^38^ The ability to engage in reflective practice to become a successful life‐long learner is therefore an approach that should be encouraged at an early stage within an undergraduate's career. It is also hoped that the use of an e‐portfolio will develop a student's digital literacy skills, a key skill required to be an effective member of the modern dental workforce.

Although the students found the asynchronous videos useful, face‐to‐face seminars would facilitate discussion of the cases between staff and students prior to the clinical session. This would also allow more opportunities for closer mentorship, particularly the development of self‐reflective and goal‐setting skills. However, the case discussions with tutors during the simulated clinic sessions did encourage students to engage in the pre‐clinical activities sufficiently to benefit from the flipped classroom style learning.^39^

Driessen^40^ states one of the elements for the successful implementation of reflective portfolios is that they should be subject to summative assessment for them to be taken seriously by students. Although the e‐portfolio workbooks were not formally assessed, they were subject to periodic monitoring and review. It could be suggested that summative assessment would have increased student engagement with the formal self‐reflection process. However, some may argue this contradicts the principle of learners as autonomous agents, and does little to develop internal self‐regulatory functions to prepare students for professional lifelong learning.^36^

This holistic approach to learning, in the absence of patients, fostered a more relaxed environment in which students felt comfortable participating in an open dialogue with clinical tutors and asking questions “in the moment” that they may have otherwise been reluctant to do so if a patient had been present. The results from both the quantitative and qualitative feedback collected through the student questionnaire showed generalised improved student confidence. Autonomy was encouraged as students devised their own diagnosis and case management, which they then compared to the suggested treatment and prevention options in the asynchronous tutor case presentation video. Decisions regarding areas such as material selection and design of prosthesis were left to the discretion of the individual, during simulated clinic sessions students would be expected to justify their proposed treatment plans and selection of materials.

Of note was the appreciation by the students of the 3D‐designed aspects of the cases and the eagerness for these to also be incorporated into future cases. In response to the qualitative feedback, investigations are currently underway for improving the materials and methods used in the production of the 3D printed teeth to more closely resemble the texture of natural enamel and dentine as well as the nature of artificial caries used in the cases. Through these means, the authors believe that confidence may be further improved through an increase in authenticity through enhanced simulation and improved tactile sensation and feedback of the material.

Another point of interest is the responses relating to confidence to “*undertake endodontic therapy*,” which received a mean rating of 3.56 out of 5. This is lower than student‐reported confidence in other clinical disciplines such as placement of direct restorations (4.48) and fixed prosthodontics (4.24). A possible explanation for this is that only one of the five simulated patient cases involved endodontic treatment, whereas five cases involved direct restorations and four fixed prosthodontics. In addition, dental students tend to perceive the field of endodontics to be more challenging.^28^, ^41^ On reflection, had more simulated patient cases involved endodontics, the results for student self‐confidence in this area may have increased.

One of the limitations of this study is the subjective nature of perceived self‐confidence. However, a separate response scale was used for questions related to confidence in an attempt to negate this. Responses to the questionnaire may potentially have suffered from elements of selection bias with those students who felt more negatively about the simulated patient cases feeling less inclined to participate, despite it being anonymous. As this study was conducted across cohorts at different stages of their undergraduate career, it is possible the variations in prior clinical experience may have influenced student responses. It should also be noted that confidence should not be conflated with student independence or clinical competence. Another limitation is that the thematic analysis was based on responses to open questions using a questionnaire, focus groups or interviews may have provided more rich data for analysis, which may be a point of further research.

Contextualisation through the use of patient histories, radiographs and photographs, provided a comprehensive background for each activity. Rather than simply undertaking isolated skill training. The results demonstrated that students felt more confident through this format of educational activity. It could be possible to expand the remit of these types of simulation activities to increase student confidence in other non‐technical skills. For example, patient videos or live, online “patient” interactions with actors could be used to help develop students' patient communication skills, which was an area not covered in the current iteration of these cases. Additional elements for incorporation include non‐clinical aspects such as complaints management, shared care with a dental therapist, consent and safeguarding.

Future case development could potentially rely quite heavily on collaboration and co‐creation with students as partners in the process. The benefits of engaging and empowering students in this way have been previously documented.^42^ At this stage of a dental undergraduate's career, a student often has a good overall grasp of the subject matter, having learnt the basic skills from experience gained during patient clinics in earlier years. As such, through reflective practice, they are uniquely placed to understand areas where knowledge may be lacking, and this could change year on year depending on cohort and developmental needs.

It has also been suggested that simulated patient cases of increasing difficulty could be incorporated into undergraduate programmes to both broaden the range of activities performed and support skills maintenance.^43^ They could also facilitate a formative standardised consistency assessment, whereby all students within a cohort would work through a case and data used to identify outliers where more learning support is required.

## 5 CONCLUSION

The learning support provided by the simulated patient cases had a positive directional relationship correlation with improved scores of student confidence and was very well received by undergraduates who believed them to be beneficial to their development. This learning intervention has the potential to improve student self‐efficacy, develop contextual competency and facilitate reflective practice. Despite this correlation with reported self‐confidence, students were not able to fully replicate the true contextual experience of delivering care to a live patient. However, simulated patient cases may be a useful tool to help close the pre‐clinical to clinical transition gap, when returning from a leave of absence during study or if a student has been identified as requiring further learning support to reach the level of a safe beginner in a pre‐clinical environment. Simulated patient cases may be a useful precursor or adjunct to traditional patient clinics. The contextualisation of simulated educational activities should be an area of further research.

## CONFLICT OF INTEREST

The authors declare that there are no conflicts of interest.

## Data Availability

The data that support the findings of this study are available on request from the corresponding author. The data are not publicly available due to privacy or ethical restrictions.
